# The coexistence of MAFLD increases fibrosis burden in patients with chronic hepatitis B

**DOI:** 10.1038/s41598-026-58144-3

**Published:** 2026-06-25

**Authors:** Alaa Mohamed Mostafa, Yasser Fouad, Nadia Abdelaaty Abdelkader, Omar Magdy, Nehal Refaat Raouf, Mohammed Eslam

**Affiliations:** 1 Department of Gastroenterology, Hepatology and Endemic Medicine, Faculty of Medicine, Minia University, Main Road, Minia, 11432 Egypt; 2 Tropical Medicine, Ain Shams University, Cairo, Egypt; 3 Department of Internal Medicine, Faculty of Medicine, Assiut University, Asyut, Egypt; 4 Department of Public Health, Faculty of Medicine, Minia University, Minya, Egypt; 5 Storr Liver Center, Westmead Institute for Medical Research, Westmead Hospital and University of Sydney, Westmead, NSW Australia

**Keywords:** Chronic hepatitis B, MAFLD, Liver fibrosis, VCTE, Diseases, Gastroenterology, Medical research

## Abstract

Despite the increasing recognition of metabolic dysfunction-associated fatty liver disease (MAFLD) and Chronic Hepatitis B (CHB), their interplay on liver fibrosis remains insufficiently elucidated. This study aimed to assess liver fibrosis in CHB patients with concurrent MAFLD. This cross-sectional study included 148 patients with a confirmed diagnosis of CHB infection, categorized according to the MAFLD standard criteria into CHB with MAFLD (CHB/+MAFLD) and without MAFLD (CHB/−MAFLD). Demographic, metabolic, and biochemical parameters were analyzed. Fibrosis was assessed non-invasively using vibration-controlled transient elastography (VCTE). A multivariate logistic regression identified independent predictors of significant fibrosis. Among the CHB cohort, 34.5% fulfilled MAFLD criteria. Using FIB-4 > 1.3 for significant fibrosis, the CHB/+MAFLD group exhibited a higher prevalence of significant fibrosis 45.1% vs. 23.7% in the CHB/−MAFLD group, with higher medians in the CHB/+MAFLD group (1.2 vs. 0.8, *p* < 0.001). Using liver stiffness measurement (LSM), those with MAFLD had a higher prevalence of significant fibrosis (F ≥ 2; 37.3 vs. 16.5%, *p* 0.005) than in those without MAFLD (medians 6.1 and 5.6 kPa, respectively, *p* 0.03). In multivariate analysis, MAFLD independently increased the odds of significant fibrosis (4.48, 95% CI 1.29–15.6, *p* 0.019) in CHB patients. The interplay of MAFLD and CHB is associated with worsening liver injury and fibrosis progression. Given the high prevalence of MAFLD and negative impact, metabolic risk assessment should be incorporated into routine CHB care to reduce fibrosis and improve long-term liver outcomes.

## Introduction

Chronic hepatitis B (CHB) infection poses a major global health challenge, affecting an estimated 296 million people worldwide, with an increasing trend of chronic liver disease cirrhosis^1,2^. Despite significant advancements in antiviral therapy, CHB remains a leading cause of liver-related morbidity and mortality, with HCC being the most serious long-term complication^3^. The worldwide prevalence of HCC remains high, especially in regions where CHB is common, directly linked to chronic infection^4^. However, factors like metabolic syndrome and obesity are also increasing causes^5,6^.

Concurrently, the worldwide rise in metabolic disorders-including obesity, type 2 diabetes, hypertension, and dyslipidemia reshaped the clinical spectrum of chronic liver disease^7,8^. Previous studies have shown a 30% of CHB patients harbor metabolically associated fatty liver affection, increasing evidence indicates that metabolic dysfunction is not only common among CHB-infected individuals but exerts a synergistic impact on liver injury and disease progression^8,9^. The metabolic–inflammatory nature of fatty liver disease was recognized as metabolic dysfunction-associated fatty liver disease (MAFLD), proposed as a more inclusive and pathophysiologically relevant framework. MAFLD defines a multisystem disorder characterized by hepatic steatosis in the presence of metabolic dysregulation, overweight/obesity, or diabetes, emphasizing its systemic and heterogeneous nature^10–13^.

While MAFLD is established as an independent driver of fibrosis in non-viral liver disease, evidence regarding its contribution to fibrogenesis in HBV infection is limited and inconsistent. The clinical interplay between MAFLD and chronic HBV infection remains insufficiently elucidated; a knowledge gap underscores the need to delineate whether concurrent MAFLD modifies the natural course of CHB-related liver disease. This study aims to assess liver fibrosis in CHB cohort patients with concurrent MAFLD.

## Patient and methods

### Study design

A cross-sectional study was conducted, including adult patients who were diagnosed with CHB infection in the outpatient clinic. Patients were recruited prospectively and consecutively during the study period (from August 2024 to August 2025), and all eligible individuals were included.

The study protocol was reviewed and approved by the institutional review board (IRB) of the Faculty of Medicine, Minia University, Minia, Egypt (Approval No. 1639). All study procedures were conducted in accordance with the Declaration of Helsinki, and written informed consent was obtained from all participants.

### Study population

The inclusion criteria for enrollment included adults (≥ 18 years) with confirmed Hepatitis B infection (HBsAg positive for ≥ 6 months) who underwent VCTE measurements. Among 179 patients examined, those with missing metabolic risk factors (*n* = 21), patients who had unreliable/invalid VCTE records (*n* = 9), and patients with dual HBV/HCV infection (*n* = 1) were excluded.

**Fig. 1:**
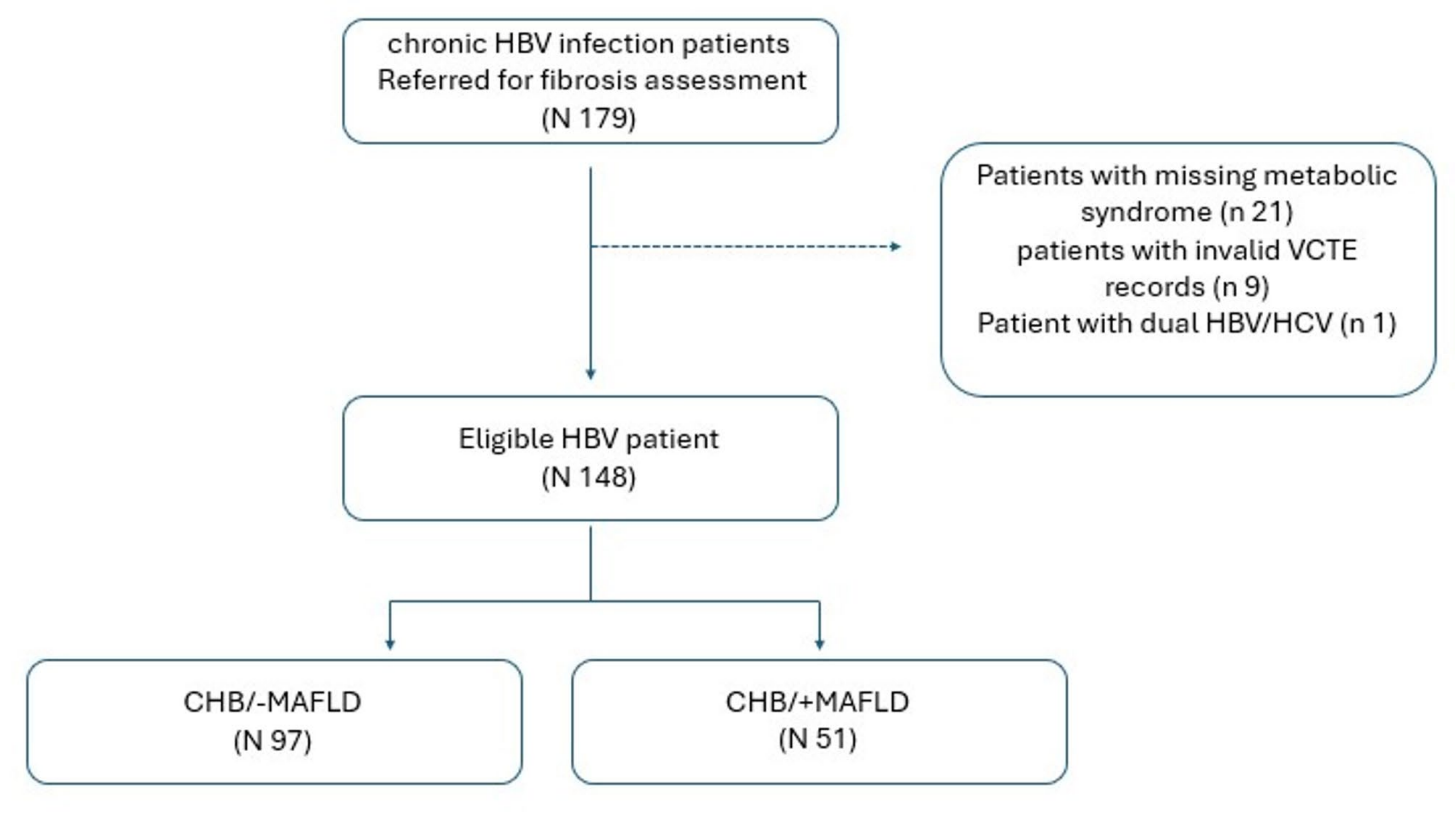
Flowchart of the HBV cohort showing patient screening, exclusions, and final stratification into CHB/−MAFLD and CHB/+MAFLD groups.

To ensure a consistent CHB cohort, patients with significant alcohol consumption or other chronic liver diseases were excluded at the outset of the study. The total number of patients enrolled in our study was 148 (Fig. 1).

### Demographic and anthropometric data

Demographic data, including age, sex, smoking, occupation, and residence, were recorded for all enrolled patients. Anthropometric measurements, including body mass index (BMI) and waist circumference (WC), were recorded for every patient. BMI was calculated by measurement of height and weight (BMI = weight (kg)/height^2^(m^2^)), and WC is measured halfway between the lowest rib and the hipbone.

### Biochemical data

Laboratory investigations included complete blood count (CBC), fasting blood sugar FBS, HbA1c, transaminases: alanine transaminase (ALT), aspartate transaminase (AST), and lipid profile (Triglycerides and Cholesterol level).

Fibrosis-4 index (FIB-4) was calculated by a standardized equation [Age*AST)/(ALT/√Platelets)]^14^, which identified significant fibrosis by a FIB-4 cut-off of ≥ 1.3.

### HBV and virologic assessment

Serum HBV DNA level was quantified as a continuous variable (IU/mL) using a standardized PCR assay.

### Definition of MAFLD

MAFLD was diagnosed in accordance with the Asian Pacific Association for the Study of the Liver (APASL) and the international expert consensus established by Eslam et al.^11,12^, through the evidence of hepatic steatosis (controlled attenuation parameter, CAP > 264 dB/m) and the presence of adiposity (overweight/obesity), diabetes (T2DM), or ≥ 2 metabolic dysregulations. Overweight is defined as a BMI ≥ 25 kg/m^2^. The history of T2DM was recorded or indicated by fasting blood glucose ≥ 126 mg/dL and/or HbA1c ≥ 6.4%. Metabolic dysregulations were assessed as follows: Hypertension is defined by elevated blood pressure ≥ 130/80 mm Hg, and current anti-hypertensive therapy was recorded. Prediabetes was evaluated by fasting blood sugar (FBS) levels of 100–125 mg/dL or HbA1c levels of 5.7–6.4%. Finally, fasting triglyceride level ≥ 150 mg/Dl or history of dyslipidemia drug.

### Assessment of fibrosis

Vibration-controlled transient elastography (FibroScan^®^, Echosens) was used to measure liver stiffness (LSM) and controlled attenuation parameter (CAP). Examinations were performed after ≥ 3 h fasting with ≥ 10 valid readings using either the M or XL probe according to body measurements, following manufacturer recommendations to ensure measurement validity. Significant fibrosis with LSM ≥ 7.2 kPa^11^.

### Statistical analysis

Data analysis was conducted using SPSS version 27. Continuous variables were expressed as median (IQR) as appropriate and compared using the Mann-Whitney U test. Categorical variables were compared using the χ^2^ test.

Multivariate logistic regression was used to identify predictors of significant fibrosis in CHB patients. Variables were selected according to clinical relevance and statistical significance in univariate analysis, with stepwise entry. Model assumptions were checked, including collinearity using variance inflation factor (VIF < 5) thresholds and tolerance values. A* p*-value < 0.05 was considered statistically significant.

## Result

### Baseline characteristics of HBV patients

A total of 148 patients with CHB were enrolled in our study. 37.1% were male, 33.1% were smokers, 23.6% were diabetic, and 21.6% were hypertensive. Anthropometric measures revealed a median BMI and waist circumference of 26.1 kg/m^2^ and 95.5 cm (Table 1).

**Table 1:** CHB patients, stratified by MAFLD criteria (CHB/−MAFLD and CHB/+MAFLD).

	All CHB (*N* = 148)	CHB/−MAFLD (*N* = 97)	CHB/+MAFLD (*N* = 51)	*p*-value
Age (years) Median (IQR)	42 (15)	40 (14)	48 (17)	0.003
Gender:				
Male	55 (37.1)	37 (38.1)	18 (35.3)	0.73
Female	93 (62.8)	60 (61.9)	33 (64.7)	
Smoking	49 (33.1)	32 (33.0)	17 (33.3)	0.96
Diabetes Mellitus	35 (23.6)	11 (11.3)	24 (47.1)	< 0.001
Hypertension	32 (21.6)	17 (17.5)	15 (29.4)	0.09
BMI (kg/m^2^)	26.1 (4.8)	25.0 (3.4)	28.8 (5.8)	< 0.001
W C (cm)	95.5 (16.5)	92 (15.2)	104 (12.0)	< 0.001
Hemoglobin (g/dL)	13.4 (1.9)	13.3 (2.0)	13.6 (1.7)	0.497
Total leukocytes (×10⁹/L)	6.6 (2.7)	6.55 (2.95)	6.80 (2.58)	0.743
Platelets (×10⁹/L)	244 (105)	260 (104)	218 (80)	0.029
ALT (U/L)	30.5 (18)	28 (20)	34 (16)	0.020
AST (U/L)	30 (16)	28 (17)	33 (15)	0.105
Triglycerides (mg/dL)	130 (81.2)	120 (66.4)	148.5 (94.6)	0.004
Cholesterol (mg/dL)	200 (76)	186 (57)	212 (75)	0.002
FBS (mg/dL)	100 (90.8)	96 (52.9)	100 (99.8)	0.099
HbA1C (%)	5.6 (1.2)	5.3 (1.1)	6.0 (1.55)	< 0.001
Fib-4	0.9 (0.7)	0.8 (0.6)	1.2 (0.6)	< 0.001
LSM (kPa)	5.8 (3.2)	5.6 (2.8)	6.1 (4.5)	0.03
PCR	1760 (14400)	1450 (6700)	5384 (17362.5)	0.1

HBV: hepatitis B virus infection; MAFLD: metabolic associated fatty liver disease; BMI: body mass index; WC: Waist circumference; ALT: alanine transferase; AST: aspartate transferase; FBS: fasting blood sugar; HbA1C: glycated hemoglobin; Fib-4: fibrosis index score; LSM: liver stiffness measurement; PCR: polymerase chain reaction.

Data analyses using χ^2^ test for categorical variables and Mann Whitney test for continuous variables.

Fifty-one (34.5%) patients fulfilled the MAFLD criteria (CHB/+MAFLD) (Fig. 1). Compared with patients with CHB alone, those with MAFLD exhibited higher medians of BMI (28.8 vs. 25 kg/m^2^) and WC (104 vs. 92 cm) (both *p* < 0.001). Concerning the biochemical data, (CHB/+MAFLD) group had lower platelet count (218 vs. 260 10*9/L, *p* 0.029), higher AST (33 vs. 28 IU/L), and ALT levels (34 vs. 28 IU/L, *P* 0.02), higher triglycerides (148.5 vs. 120 mg/dL, *p* 0.004), cholesterol (212 vs. 186 mg/dL, *p* 0.002), and HbA1C (6 vs. 5.3%, *p* < 0.001) levels than those CHB/−MAFLD. Median PCR levels showed no significant difference between our studied CHB patients with and without MAFLD.

### Assessment of fibrosis in HBV patients

**Fig. 2:**
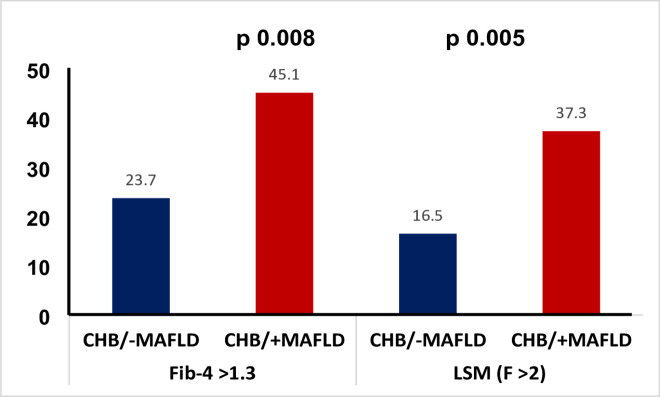
Significant fibrosis in CHB with and without MAFLD (Comparing significant fibrosis between CHB/−MAFLD and CHB/+MAFLD groups, using FIB-4 and LSM).

Non-invasive fibrosis indices varied between groups. Using FIB-4 > 1.3 as a threshold for significant fibrosis, the CHB/+MAFLD group exhibited a higher prevalence of significant fibrosis (45.1%) than the CHB/−MAFLD group (23.7%), with higher medians in the CHB/+MAFLD group (1.2 vs. 0.8, *p* < 0.001) (Fig. 2).

Consistently, liver stiffness measurement (LSM) assesses fibrosis severity. The prevalence of significant fibrosis (F ≥ 2) was higher in CHB/+MAFLD (37.3 vs. 16.5%, *p* 0.005) than in those without MAFLD (with medians 6.1 and 5.6 kPa, respectively, *p* 0.03).

### Risk factors of significant fibrosis in CHB patients

When stratified by fibrosis status, 23.6% of CHB patients had significant fibrosis (F ≥ 2) (Table 2). CHB/+fibrosis patients were older (49 vs. 40 years, *p* 0.004), had higher BMI (27.8 vs. 26 kg/m^2^, *p* 0.005) and higher WC (103.6 vs. 95 cm). Diabetes was more prevalent in the fibrosis group (37.1% vs. 19.5%, *p* 0.032), associated with higher HbA1c (6.1 vs. 5.3%, *p* 0.001), lower platelets count (209 vs. 250 10*9, *p* 0.006), higher Triglyceride (138.5 vs. 120 mg/dL, *p* 0.01) and Cholesterol (223.5 vs. 190 mg/dL, *p* 0.04). The serum transaminases were elevated in the presence of fibrosis (ALT 37 vs. 28 U/L, *p* 0.039 and AST 38 vs. 27 U/L, *p* 0.002). Mean platelet counts were lower in advanced fibrosis (*p* 0.006). The prevalence of MAFLD was higher in the HBV group with significant fibrosis (54.3% vs. 28.3%, *p* 0.005).

Moreover, in the Multivariate regression model, assessing risk factors of significant fibrosis in CHB patients, MAFLD showed 4.48 odds of having significant fibrosis (CI 1.29–15.6, *p* 0.019), while ALT (OR: 1.06; CI 1.01–1.10, *p* 0.01) and male gender (female OR: 0.18; 95% CI 0.05–0.68, *p* 0.012) were considered predictors for significant fibrosis in HBV patients (Table 3). While age (OR: 1.4; 95%CI 0.98–1.09, *p* 0.137), platelet count (OR: 0.99; 95%CI 0.99–1.00, *p* 0.281), triglycerides (OR: 1.34; 95%CI 0.36–5.0 *p* 0.66), and HbA1c (OR: 1.31; 95%CI 0.85–2.00, *p* 0.233), did not reach statistical significance in the final model. The overall regression model demonstrated good fit, with deviance of 81.4, AIC of 97.4, McFadden’s R^2^ of 0.272, and a likelihood ratio χ^2^ of 30.4 (df = 7, *p* < 0.001).

**Table 2:** CHB patients stratified by LSM (F ≥ 2, CHB/-Fibrosis, and CHB/+Fibrosis).

	CHB/-Fibrosis (*N* = 113)	CHB/+Fibrosis (*N* = 35)	*p*-value
Age Median (IQR)	40 (13)	49 (20.5)	0.004
Gender			
Male	37 (32.7)	18 (51.4)	0.046
Female	76 (67.3)	17 (48.6)	
Smoking	35 (31.0)	14 (40.0)	0.321
BMI (kg/m^2^)	26 (4.4)	27.8 (7.7)	0.005
W C (cm)	95 (13.9)	103.6 (21.7)	0.111
Diabetes mellitus	22 (19.5)	13 (37.1)	0.032
Hypertension	21 (18.6)	11 (31.4)	0.107
Platelet count (×10⁹/L)	250 (91)	209 (98)	0.006
ALT (U/L)	28 (16)	37 (30)	0.039
AST (U/L)	27 (14)	38 (31)	0.002
Triglyceride (mg/dL)	120 (68.7)	138.5 (108.2)	0.01
Cholesterol (mg/dL)	190 (48.5)	223.5 (82)	0.040
FBS (mg/dL)	97 (92.7)	100 (18)	0.045
HbA1C (%)	5.3 (1)	6.1 (1.8)	0.001
MAFLD	32 (28.3)	19 (54.3)	0.005

MAFLD: metabolic-associated fatty liver disease; BMI: body mass index; WC: Waist circumference; ALT: alanine transferase; AST: aspartate transferase; FBS: fasting blood sugar; HbA1C: glycated hemoglobin; CAP: controlled attenuated parameter.

Data analyses using χ^2^ test and Mann Mann-Whitney test for categorical and continuous variables.

**Table 3:** Predictors of significant fibrosis in patients with CHB infection.

	95% CI for OR	*p*-value
Age	1.4 (0.98–1.09)	0.137
Gender: Female	0.18 (≈ 0.05–0.68)	0.012
Platelets (×10^9^/L)	0.99 (≈ 0.99–1.00)	0.281
ALT (U/L)	1.06 (≈ 1.01–1.10)	0.010
Triglyceride (mg/dL)	1.34 (≈ 0.36–5.0)	0.660
HbA1C (%)	1.31 (≈ 0.85–2.00)	0.233
MAFLD	4.48 (≈ 1.29–15.6)	0.019

MAFLD: metabolic-associated fatty liver disease; ALT: alanine transferase; HbA1C: glycated hemoglobin.

## Discussion

In this prospective cross-sectional study, we assess the impact of MAFLD on a cohort of 148 CHB patients. A third of those patients fulfilled the diagnostic criteria for MAFLD (CHB/+MAFLD) and exhibited significantly higher metabolic and biochemical risk profiles than those without MAFLD (CHB/−MAFLD). Likewise, the coexistence of MAFLD was linked with a higher prevalence and severity of significant fibrosis in patients with CHB infection, as far that MAFLD could be identified as an independent predictor of significant fibrosis in CHB patients, highlighting the impact of integrating MAFLD criteria for risk-stratification of liver fibrosis in CHB patients.

MAFLD was previously discussed with a reported pooled prevalence range of 29–35% in HBV patients^15–17^. In a previous meta-analysis, metabolic components, including diabetes, adiposity, and dyslipidemia, were identified as the main determinants of fibrosis-related adverse outcomes rather than viral parameters in CHB patients^9,18^. In patients with concurrent MAFLD and CHB, we found a higher liver injury and Fibrosis indices compared with their counterparts without MAFLD. This was aligned with previous histological and elastography-based studies showing that metabolic dysfunction potentiates necroinflammation and fibrogenesis in CHB through synergistic activation of lipotoxic, oxidative, and inflammatory pathways^19–21^. The metabolic-viral interaction was previously discussed; supposed that metabolic dysfunction, including insulin resistance, adipokine imbalance, and lipid accumulation, aggravates fibrosis in CHB patients^10,16^. Besides the notion that MAFLD modifies disease development through metabolic–fibrotic coupling, irrespective of viral replication, suggesting a metabolically driven hepatopathy phenotype in HBV infection^10^.

In our CHB cohort, we found that patients CHB/+MAFLD were more likely to exhibit higher degrees of significant fibrosis compared to those without; in addition, MAFLD remained an independent predictor of fibrosis in CHB patients. Our findings suggest that metabolic dysfunction substantially associated with fibrogenic risk in those patients, consistent with evidence that the metabolic milieu drives fibrosis progression beyond viral effects^21^. Furthermore, in biopsy-proven studies, steatohepatitis, or concurrent fatty liver in CHB, was linked with greater fibrosis stage and necroinflammatory activity^20,22^. Along with a previous study using transient elastography, hepatic injury and fibrosis positively correlated with CAP values in HBV, supporting a metabolic–fibrotic axis^23^.

In our study, MAFLD demonstrated more than 4-fold higher odds of significant fibrosis among chronic HBV patients, reinforcing the concept that metabolic stressors, not viral load, drive fibrogenesis in the HBV–MAFLD overlap. Comparable studies reported that metabolic comorbidities, especially diabetes and central obesity, doubled to quadruple the odds of cirrhosis and hepatocellular carcinoma among treated HBV patients^24–26^. Furthermore, we found HBV DNA levels did not differ between patients with and without MAFLD, indicating no direct relationship between viral replication and metabolic-associated fatty liver disease. Aligned with accumulating evidence showing that MAFLD in HBV patients is metabolically rather than virologically driven, reported a diversity of inverse or neutral associations between HBV activity and steatosis^27–29^, emphasizing metabolic dominance over virological determinants.

The mechanisms of the metabolic-fibrotic injury in the HBV/MAFLD overlap are multifactorial. Lipid accumulation and insulin resistance increase oxidative stress and hepatocyte injury, thereby exacerbating necroinflammation and fibrogenesis^8,13^. Furthermore, imbalance of adipokines, such as altered leptin and adiponectin signaling, contributes to the activation of hepatic stellate cells and progression of fibrosis^16,17^. Likewise, inflammatory mediators like TNF-α and IL-6 potentiate liver injury, supporting the notion of a metabolic-fibrotic axis that operates independently of viral replication^18,20^. Consequently, these pathways illustrate how metabolic dysfunction synergizes with HBV infection to drive fibrosis, underscoring the critical need for integrated metabolic and antiviral management strategies^7,21^.

This study has several limitations. First, its cross-sectional design and modest sample size preclude causal inference regarding the relationship between MAFLD and fibrosis progression in chronic HBV, while also limiting longitudinal follow-up for disease progression. Second, the diagnosis of MAFLD relied on available metabolic and biochemical parameters rather than histologic confirmation. Additionally, HBV viral load activity was captured by a single-point PCR measurement, which may not accurately reflect chronic viral dynamics or treatment status. Additional HBV-related clinical characteristics, including antiviral therapy status, HBeAg status, duration of infection, and disease stage were not uniformly available in our cohort and therefore could not be incorporated into the analysis, and could potentially impact fibrosis outcomes. Finally, a single center-derived cohort, potentially limiting external generalizability to broader populations with different ethnicities, genetic, and metabolic profiles. Despite these limitations, the consistent association observed between MAFLD and fibrosis across multiple non-invasive measures supports the robustness of our findings.

In conclusion, our study demonstrated a synergistic relationship between CHB and MAFLD, indicating worsened metabolic dysfunction and hepatic fibrosis advancement. This finding supports the paradigm that MAFLD-related metabolic steatosis stressors dictate disease trajectory in CHB. A metabolic-fibrotic phenotype within CHB that warrants targeted metabolic screening and management alongside antiviral therapy. Further workup is needed to improve precision hepatology methods and strengthen the MAFLD framework as a key part of HBV disease classification and long-term prognosis. Future studies would benefit from employing prospective, multicenter designs with diverse ethnic populations to validate these findings and clarify long-term causal relationships. Further clinical trials exploring combined antiviral and metabolic treatment strategies are suggested to determine the best management approaches.

## Data Availability

The data analyzed in this study are owned by the authors and are available upon request due to ethical considerations.

## References

[1] Hsu, Y. C., Huang, D. Q. & Nguyen, M. H. Global burden of hepatitis B virus: Current status, missed opportunities and a call for action. *Nat. Rev. Gastroenterol. Hepatol.* **20** (8), 524–537. 10.1038/s41575-023-00760-9 (2023). 37024566 10.1038/s41575-023-00760-9

[2] Cao, G., Jing, W., Liu, J. & Liu, M. Countdown on hepatitis B elimination by 2030: The global burden of liver disease related to hepatitis B and association with socioeconomic status. *Hepatol. Int.* **16** (6), 1282–1296. 10.1007/s12072-022-10410-y (2022). 36048317 10.1007/s12072-022-10410-y

[3] Singh, S. P., Madke, T. & Chand, P. Global epidemiology of hepatocellular carcinoma. *J. Clin. Exp. Hepatol.* **15** (2), 102446. 10.1016/j.jceh.2024.102446 (2025). 39659901 10.1016/j.jceh.2024.102446 PMC11626783

[4] Khattab, M. A., Eslam, M., Sharwae, M. A. & Hamdy, L. Seroprevalence of hepatitis C and B among blood donors in Egypt: Minya Governorate, 2000–2008. *Am. J. Infect. Control*. **38** (8), 640–641. 10.1016/j.ajic.2009.12.016 (2010). 20400204 10.1016/j.ajic.2009.12.016

[5] Wang, X. et al. Antiviral therapy reduces mortality in hepatocellular carcinoma patients with low-level hepatitis B viremia. *J. Hepatocell. Carcinoma* **21** (8), 1253–1267. 10.2147/JHC.S330301 (2021). 10.2147/JHC.S330301 PMC8544274 34708007

[6] Yang, J. D. et al. A global view of hepatocellular carcinoma: Trends, risk, prevention and management. *Nat. Rev. Gastroenterol. Hepatol.* **16** (10), 589–604. 10.1038/s41575-019-0186-y (2019). 31439937 10.1038/s41575-019-0186-y PMC6813818

[7] Vargas, M. et al. Metabolic disease and the liver: A review. *World J. Hepatol.* **16** (1), 33–40. 10.4254/wjh.v16.i1.33 (2024). 38313243 10.4254/wjh.v16.i1.33 PMC10835488

[8] Pan, Z. et al. The burden of metabolic diseases in the Arab region, 1990–2021. *Ther. Adv. Endocrinol. Metab.* **16**, 20420188251406531. 10.1177/20420188251406531 (2025). 41425688 10.1177/20420188251406531 PMC12715173

[9] Huang, S. C. & Liu, C. J. Chronic hepatitis B with concurrent metabolic dysfunction-associated fatty liver disease: Challenges and perspectives. *Clin. Mol. Hepatol.* **29** (2), 320–331. 10.3350/cmh.2022.0422 (2023). 36726053 10.3350/cmh.2022.0422 PMC10121303

[10] Eslam, M. et al. A new definition for metabolic dysfunction-associated fatty liver disease: An international expert consensus statement. *J. Hepatol.* **73** (1), 202–209. 10.1016/j.jhep.2020.03.039 (2020). 32278004 10.1016/j.jhep.2020.03.039

[11] Eslam, M. et al. The Asian Pacific association for the study of the liver clinical practice guidelines for the diagnosis and management of metabolic dysfunction-associated fatty liver disease. *Hepatol. Int.* **19** (2), 261–301. 10.1007/s12072-024-10774-3 (2025). 40016576 10.1007/s12072-024-10774-3

[12] Eslam, M., Sanyal, A. J. & George, J. International consensus panel. MAFLD: A consensus-driven proposed nomenclature for metabolic associated fatty liver disease. *Gastroenterology* **158** (7), 1999–2014. e1 (2020). 32044314 10.1053/j.gastro.2019.11.312

[13] Fouad, Y. et al. The African Middle East Association of Gastroenterology (AMAGE) clinical practice guidelines for the diagnosis and management of metabolic dysfunction associated fatty liver disease. *Ann. Hepatol.* **31** (1), 102180. 10.1016/j.aohep.2026.102180 (2026). 41520698 10.1016/j.aohep.2026.102180

[14] Vallet-Pichard, A. et al. FIB-4: An inexpensive and accurate marker of fibrosis in HCV infection. Comparison with liver biopsy and fibrotest. *Hepatology* **46** (1), 32–36. 10.1002/hep.21669 (2007). 17567829 10.1002/hep.21669

[15] Machado, M. V., Oliveira, A. G. & Cortez-Pinto, H. Hepatic steatosis in hepatitis B virus infected patients: Meta-analysis of risk factors and comparison with hepatitis C infected patients. *J. Gastroenterol. Hepatol.* **26**, 1361–1367. 10.1111/j.1440-1746.2011.06801 (2011). 21649726 10.1111/j.1440-1746.2011.06801.x

[16] Zheng, Q. et al. Systematic review with meta-analysis: Prevalence of hepatic steatosis, fibrosis and associated factors in chronic hepatitis B. *Aliment. Pharmacol. Ther.* **54**, 1100–1109. 10.1111/apt.16595 (2021). 34469587 10.1111/apt.16595

[17] Jiang, D. et al. Concurrence and impact of hepatic steatosis on chronic hepatitis B patients: A systematic review and meta-analysis. *Ann. Transl Med.* **9**, 1718. 10.21037/atm-21-3052 (2021). 35071412 10.21037/atm-21-3052 PMC8743703

[18] Zhu, Q. et al. Impact of metabolic dysfunction-associated fatty liver disease of varying severity on antiviral treatment outcomes and clinical prognosis in patients with chronic hepatitis B: A systematic review and meta-analysis. *Infect. Dis. Ther.* **14** (8), 1599–1617. 10.1007/s40121-025-01189-0 (2025). 40638020 10.1007/s40121-025-01189-0 PMC12339849

[19] Seto, W. K. et al. Association between hepatic steatosis, measured by controlled attenuation parameter, and fibrosis burden in chronic hepatitis B. *Clin. Gastroenterol. Hepatol.* **16** (4), 575–583e2. 10.1016/j.cgh.2017.09.044 (2018). 28970146 10.1016/j.cgh.2017.09.044

[20] Choi, H. S. J. et al. Nonalcoholic steatohepatitis is associated with liver-related outcomes and all-cause mortality in chronic hepatitis B. *Hepatology* **71**, 539–548. 10.1002/hep.30857 (2020). 31309589 10.1002/hep.30857

[21] Lv, H. et al. Liver fibrosis is closely related to metabolic factors in metabolic associated fatty liver disease with hepatitis B virus infection. *Sci. Rep.* **13** (1), 1388. 10.1038/s41598-023-28351-3 (2023). 36697471 10.1038/s41598-023-28351-3 PMC9877001

[22] Chan, A. W. et al. Concurrent fatty liver increases risk of hepatocellular carcinoma among patients with chronic hepatitis B. *J. Gastroenterol. Hepatol.* **32** (3), 667–676. 10.1111/jgh.13536 (2017). 27547913 10.1111/jgh.13536

[23] Qi, X. et al. Transient elastography for significant liver fibrosis and cirrhosis in chronic hepatitis B: A meta-analysis. *Can. J. Gastroenterol. Hepatol.* **2018**, 3406789. 10.1155/2018/3406789 (2018). 29977884 10.1155/2018/3406789 PMC5994263

[24] Fan, R. et al. Association of central obesity with hepatocellular carcinoma in patients with chronic hepatitis B receiving antiviral therapy. *Aliment. Pharmacol. Ther.* **54** (3), 329–338. 10.1111/apt.16469 (2021). 34157146 10.1111/apt.16469

[25] Pan, Z. et al. Changing profile and burden of hepatocellular carcinoma in Arab Countries in 1990–2021. *Ann. Hepatol.* **30** (2), 102104. 10.1016/j.aohep.2025.102104 (2025 Jul-Dec). 10.1016/j.aohep.2025.102104 40882815

[26] Bockmann, J. H. et al. High rates of liver cirrhosis and hepatocellular carcinoma in chronic hepatitis b patients with metabolic and cardiovascular comorbidities.* Microorganisms*** 9**(5), 968. 10.3390/microorganisms9050968 (2021). 10.3390/microorganisms9050968 PMC8146494 33946154

[27] Hui, R. W. H. et al. Inverse relationship between hepatic steatosis and hepatitis B viremia: Results of a large case-control study. *J. Viral Hepat.* **25** (1), 97–104. 10.1111/jvh.12766 (2018). 28772340 10.1111/jvh.12766

[28] Mak, L. Y. et al. Diverse effects of hepatic steatosis on fibrosis progression and functional cure in virologically quiescent chronic hepatitis B. *J. Hepatol.* **73** (4), 800–806. 10.1016/j.jhep.2020.05.040 (2020). 32504663 10.1016/j.jhep.2020.05.040

[29] Wang, M. M. et al. Hepatic steatosis is highly prevalent in hepatitis B patients and negatively associated with virological factors. *Dig. Dis. Sci.* **59** (10), 2571–2579. 10.1007/s10620-014-3180-9 (2014). 24838496 10.1007/s10620-014-3180-9

